# Anxious and Angry: Early Emotional Adaptation of Medical Students in a Situational Crisis on the Example of the COVID-19 Pandemic

**DOI:** 10.3390/ijerph20031847

**Published:** 2023-01-19

**Authors:** Julia Wyszomirska, Monika Bąk-Sosnowska, Anna Daniel-Sielańczyk

**Affiliations:** 1 Department of Psychology, Faculty of Health Sciences in Katowice, Medical University of Silesia in Katowice, 40-752 Katowice, Poland; 2 Center for Psychosomatics and Health Prevention, WSB University in Dąbrowa Górnicza, 41-300 Dąbrowa Górnicza, Poland

**Keywords:** anxiety, anger, medical students, crisis situations, emotional state, COVID-19

## Abstract

Background: The COVID-19 pandemic is an example of a situational crisis resulting in emotional destabilization. The aim of the study was to analyze changes in the level of anxiety and anger in medical students during the early adaptation to the situational crisis, and to estimate the risk factors for fear and anger in this group. Methods: Participants were 949 medical students (M = 22.88, SD = 4.10) in the first stage on March 2020, and 748 (M = 22.57, SD = 3.79) in the second stage on June 2020. The STAI, STAXI-2, and our own questionnaire were used. Results: First vs the second stage: anxiety state (*p* < 0.001), anger state (*p* = 0.326), and feeling angry (*p* < 0.05). The regression model (F(14.1681) = 79.01, *p* < 0.001) for the level of anxiety state explains 39% of the dependent variable variance (*r*^2^ = 0.39). The model for the anger-state level (F(6.1689) = 68.04, *p* < 0.001)-19% (*r*^2^ = 0.19). Conclusions: During the early adaptation to the situational crisis, the general level of anxiety decreased, but anger was at the same level. The anxiety was explained by contact with potentially or objective infected persons, and the level of anger was based on the need for greater social support.

## 1. Introduction

A psychological crisis is a reaction to circumstances that are difficult to overcome. These are specific, unexpected, and non-routine events or series of events that create a high level of uncertainty and threaten the achievement of important goals. They are associated with negative emotions, a lack of resources for effective coping, and difficulties in optimal functioning [1]. Unforeseeable crises, such as accidents, disasters, or losses, are called situational crises. Situations included in this group are sudden events that occur during an individual’s life. Their course is often violent and traumatic due to the element of surprise that accompanies such events. Compared with developmental crises, situational ones are usually shorter in duration, although their consequences can be long-lasting, often changing a person’s life forever.

According to the cognitive theory of crisis, the response to the crisis depends not only on the specificity of the event but, above all, on the subjective interpretation of the individual. It is expressed in a specific perception (cognitive aspect) or emotional reaction (affective aspect), which is the basis of crisis behavior (behavioral aspect) [2]. An individual in a mental crisis may display negative emotions (e.g., anger, regret, fear, sadness, fear, rage, despair, helplessness, loneliness, helplessness, guilt, shame, hope, and doubt), negative beliefs (e.g., pessimistic, related to helplessness), and varied behaviors (from numbness and withdrawal to fighting and seeking help). People may react inadequately to the situation, abuse psychoactive substances, and even pose a threat to themselves or others [3]. At the same time, despite all the dramatic symptoms and consequences of a situational crisis, it can also contribute to a person’s mental, emotional, or spiritual development under certain circumstances. Post-traumatic growth is favored by, among others, a focus on the problem, acceptance and positive reinterpretation of the event, optimism, religion, and positive affect [4].

Epidemics of infectious diseases are an example of a situational crisis on a massive scale. Over the centuries, humanity has struggled with them many times, as in the cases of plague, cholera, typhus, smallpox, syphilis, leprosy, or flu [5]. In the 20th century, the flu spread three times on an enormous scale. Beginning in 1918 as the so-called Spanish flu, and then in 1957 as the Hong Kong Asian flu, and in 2009 as the swine flu. The considerable spread of these diseases was due to the mobility of people on a scale that had never occurred before. This also applies to the current COVID-19 pandemic [6]. The COVID-19 infectious disease caused by the SARS-CoV-2 coronavirus began as an epidemic on 17 November 2019, in the city of Wuhan, Hubei province, central China, and was recognized by the World Health Organization (WHO) on 11 March 2020 as a pandemic [7]. By 2 November 2022, 636,125,357 cases of SARS-CoV-2 coronavirus infection were reported worldwide, of which 6,595,987 people died [8].

In Poland, the first case of SARS-CoV-2 infection was reported on 4 March 2020. On 12 March 2020, by the decision of the Minister of Science and Higher Education, classes were suspended at Polish universities and it was recommended to implement online classes [9]. On 20 March 2020, the Polish government introduced the state of the epidemic, limiting leaving home, free movement, and gatherings of more than two people [10]. A period of social deprivation began, additionally marked by a sense of uncertainty, fear, and the threat of potential infection and disease. This period was particularly difficult for pupils and students who, due to the specificity of adolescence, need contact with peers, direct communication, cooperation, and social acceptance. Studies have shown that social deprivation during adolescence affects brain function and can have far-reaching consequences related to functioning in adulthood [11].

Only from 30 May 2020, the fourth and final stage of lifting restrictions began in Poland. Limits on people in the commercial and catering industry were removed. Cinemas, theatres, operas, swimming pools, fitness clubs, playgrounds and amusement parks, saunas, and solariums were resumed from 6 June 2020 [12]. Although the state of the epidemic was still in force, the situation regarding social contact changed significantly, despite the need to cover your mouth and nose in public spaces. The period of almost three months that passed from the introduction of remote learning to the lifting of the ban on direct social contact can be considered a time of early adaptation to a situational crisis. For energy reasons, the human body and psyche cannot function in total mobilization for an infinitely long time. Therefore, after some time, an acute crisis, if not positively ended, turns into a chronicle crisis, and a relative adaptation to the new situation takes place. It usually happens after a maximum of 6 to 8 weeks [13]. Psychological adaptation is a process anchored in emotions and intellect, owing to which people maintain balance in their mental and emotional states and interactions with the social and cultural environment [14]

The situation of a pandemic, uncertainty about the future, disinformation, and fear for one’s own and loved ones’ lives may contribute to the emergence of difficult emotions, increased levels of stress and tension, and thus the occurrence of anxiety and depressive disorders [15]. In addition, the disclosure of anger, which, along with fear, is the primary reaction of an individual to a violation of personal security. Despite experiencing these unpleasant emotions, they can play an adaptive role because the threat mobilizes the individual to an increased effort related to fighting or flight [16]. In turn, prolonged high levels of anxiety and anger increase susceptibility to disease, weaken the immune system, raise lipid levels, increase pain, and increase the risk of death from cardiovascular disease and all other causes [17]. While the reactions of fear and stress in pandemic situations are well researched [18,19], anger is much less so [20]. At the same time, the presence of these negative emotions is well documented in the medical staff group and is understandable due to the risk of infection and work overload during pandemic periods [21,22]. At the same time, the data on future medical workers, i.e., students of medical faculties, are much poorer [23,24]. Their situation differs from employees mainly in the stage of psychosocial development and the scope of individual needs, but also the consequences of possible involvement in work with COVID-19 patients. On the one hand, there may be serious concerns (risk of infection, insufficient time to learn). On the other hand, the responsibility is incomparably less, and the potential benefits of unique clinical experience are significant.

The authors of this work, as academic teachers in medical faculties, therefore set themselves the following research question: What are the dynamics of changes in the emotional state of students during the first months of the pandemic, and what factors have the greatest impact on their emotional wellbeing? While testing the level of anxiety in a situational crisis is common, we wanted to fill the gap in the field of anger reactions. In addition to enriching psychological knowledge, we wanted to use the results to plan psychological intervention and supplement educational programs with elements preparing students for potential situational crises. Therefore, our research aimed to analyze changes in the level of anxiety and anger in medical students during the early adaptation to the situational crisis in the example of the COVID-19 pandemic, as well as to estimate the risk factors for high levels of fear and anger in this group.

## 2. Materials and Methods

### 2.1. Participants

The inclusion criteria were a minimum age of 18, declaration of currently studying at the Medical University of Silesia in Katowice (MUS), and confirmation of informed consent to participate in the study. Apart from withdrawal from the study, no other exclusion criteria were adopted. Finally, 1697 students, aged 19 to 56, participated in the study. Most of them were studying medicine (n = 629, 37.06%) and then nursing (n = 204, 12.02%), physiotherapy (n = 201, 11.84%), pharmacy (n = 161, 9.48%), dentistry (n = 148, 8.72%), midwifery (n = 104, 6.12%), medical analytics (n = 85, 5.00%), medical biotechnology (n = 69, 4.06%), and dietetics (n = 58, 3.41%). The remaining participants (n = 38, 2.23%) were electroradiology, cosmetology, emergency medical services, medical coaching, public health, and neurobiology students. This distribution is adequate for the actual number of students in particular fields of study at MUS. The stage I (I) of the study began on 20 March 2020, i.e., the day of the announcement of the epidemic state in Poland [10] and lasted until 2 April 2020. A total of 949 students participated (M = 22.88; SD = 4.10), constituting 9.24% of all SUM students in 2020 (n = 10,267). The stage II (II)—the next measurement in the same population—was carried out after three months, i.e., in the period from 8 June to 31 July, 2020. A total of 748 people (M = 22.57; SD = 3.79) took part in it, accounting for 7.28% of the studied student population. Women dominated in both stages. There were no statistically significant differences in the age and sex of the subjects in both stages. Most of the respondents (I: 67.5%; II: 68.4%) claimed that in the last 14 days they had no contact with people diagnosed or suspected of COVID-19. A total of 77.3% (I) and 72.7% (II) indicated no health problems. At each stage of the study, 0.8% of people confirmed that they were currently undergoing immunosuppressive treatment, and 4.7% were diagnosed with reduced immunity. Table 1 presents the sociodemographic data and data on chronic diseases and current symptoms that may indicate COVID-19, both for the 1st and 2nd measurements.

Generally, respondents assessed their general health as good or very good (I: n = 783, 82.5%; II: n = 586, 78.3%). Those who confirmed current symptoms suggesting COVID-19 usually reported one or two symptoms (I: n = 434, 45.7%; II: n = 286, 38.2%). A total of 11 (1.15%) surveyed in stage I and 13 (1.73%) surveyed in stage II benefited from professional psychological support due to the pandemic situation. In turn, 344 (36.2%) in stage I and 247 (33.0%) in stage II indicated that they needed more than usual support from other people. At the same time, 69.1% (n = 656) in stage I and 66.6% (n = 498) in stage II assessed the level of received support as sufficient. The remaining participants were not satisfied with the level of received support. According to 25.1% (n = 276) in stage I and 31.9% (n = 239) in stage II, it was too little or none. Additionally, 142 persons (14.9%) in stage I confirmed their involvement in volunteering activities related to the COVID-19 pandemic, while in stage II there were no such people. Generally, the respondents were not convinced about the possibility of receiving adequate medical care in the case of falling ill with COVID-19. Most frequently, both in stage I (n = 66, 69.8%) and stage II (n = 509, 68.0%), participants assessed these chances as ranging from minimal to average.

### 2.2. Study Organization

The research is based on the data obtained in repeated two cross-sectional studies (stages I and II) performed in the same population—students of the Medical University of Silesia in Katowice. Therefore, it includes two groups of students tested in two consecutive research periods. All persons with the status of a student of one of the 5 SUM departments in Katowice were invited to fill out online anonymous questionnaires for each of the stages of the study through the university e-mail, the SUM website, or university FB websites.

### 2.3. Ethics

Participation in the study was voluntary and anonymous. The consent to participate in the study was part of the questionnaire. Each of the potential respondents obtained information about the purpose and method of the study. There was no payment for participation. The study was approved by the relevant Bioethical Committee (approval No. PCN/0022/KB/59/20). The authors designed and conducted the study in compliance with the applicable provisions of law and followed in accordance with the ethical guidelines of the Declaration of Helsinki as revised in 2013.

### 2.4. Methods

We used the diagnostic survey method. Standardized psychological tests were used to assess the severity of the two main psychological variables—the state of anxiety and anger.

The State–Trait Anger Expression Inventory—STAXI-2 for condition, trait, expression, and anger control by C.D. Spielberger in the Polish adaptation of W. Bąk was used [25]. The first of three parts investigating the state of anger was used—State Anger. It consists of 15 items and measures the intensity of anger at a given moment in time. The subscales Feeling Angry (F), Feeling Like Expressing Anger Verbally (V), and Feeling Like Expressing Anger Physically (P) are distinguished. The person is asked to respond to the given statements on a scale from 1 (definitely not) to 4 (definitely yes). The measurement reliability estimated with Cronbach’s alpha coefficient ranged from 0.90 to 0.93 depending on subscales and overall State Anger was the highest α = 0.96.

The State–Trait Anxiety Inventory for Adults—STAI, C.D. Spielberger, R.L. Gorsuch, and R.E. Lushene in the Polish adaptation of C.D. Spielberger, J. Strelau, M. Tysarczyk, and K. Wrześniewski was used [26]. In the study, the X-1 subscale was used to measure state anxiety treated as a transient and situational state of the individual. The X-1 subscale consists of 20 items to which the respondent answers by selecting one of 4 categorized responses (not at all, somewhat, moderately so, or very much so). Reliability in our own study was α = 0.95.

A self-survey questionnaire was also used to collect demographic and clinical data, beliefs related to the pandemic situation, and behaviors undertaken to reduce the risk of virus transmission. Based on the questions of the survey, three indicators were developed and used for further statistical analysis. The internal consistency of the indicators described below is satisfactory.

The subjective assessment of the risk of relatives becoming seriously ill in the case of a possible infection with COVID-19 is reflected by the average response to the above-mentioned questions about parents, grandparents, partners, siblings, children, and friends of the respondents. The Cronbach’s alpha coefficient for this scale, consisting of 6 items, is 0.82.

Adherence to recommendations to reduce the risk of infection of oneself and/or others was created by summing up the answers to 13 questions including: avoiding touching the eyes, nose, and mouth; covering the mouth when sneezing or coughing with an elbow or tissue; washing hands; disinfecting hands and objects; keeping a minimum distance of 1.5 m from people in public spaces; avoiding public places and avoiding leaving home without a clear need; limiting direct contact with loved ones; avoiding the use of shared dishes during meals; avoiding greeting by shaking hands; wearing a protective mask; preferring to work/learn online rather than to work/learn stationary or by keeping the recommended social distance. The value of Cronbach’s alpha for this scale is 0.84.

The indicator of adequate potential actions to be taken in the case of contracting the infection was created by the mean of the correct answers to 10 questions including: no action; wearing a protective mask; staying home for at least 14 days; informing people about the infection if there was a contact with them; telephone contact with the primary care physician; telephone contact with State Sanitary Inspection/National Health Service (pl. Narodowy Fundusz Zdrowia); telephone contact with the infectious diseases ward/hospital; personally reporting to a primary healthcare physician; personally reporting to the emergency room; and personally reporting to the infectious diseases ward/hospital. For the correctness index of predicting behaviors, the reliability of the measurement was not calculated, as it was an index of knowledge, so the answers of the respondents did not have to be consistent.

In addition, we asked for a subjective assessment with regard to:

The risk of infection, which was based on answers to 3 questions on a 5-point Likert scale regarding the risk of: getting infected; transferring the virus to other people; or becoming seriously ill due to infection.

The need for social support was assessed based on a dichotomous answer to the question: “Have I needed more support from other people than usual over the last 14 days?”. We also asked for an assessment of the level of adequacy of support received and provided to others: none, insufficient, sufficient, or too much.

The subjective level of stress was assessed by answering a question on a 5-point Likert scale: “Try to assess how stressful the coronavirus epidemic is for you”.

### 2.5. Statistics

To verify the research hypotheses, we carried out statistical analyzes using the IBM SPSS Statistics 25 software. Using this tool, we performed frequency analyses, basic descriptive statistics analyses together with the Kolmogorov–Smirn ov test, analysis correlation with Pearson’s correlation coefficient, Spearman’s rank correlation, and Student’s *t*-test for independent samples. The standard significance level α = 0.05 was adopted. In the case of distributions other than normal, the value of the skewness of the distribution of the examined variables was verified. When it fell within the range of +/−2, it was assumed that the distribution of the studied variable was not significantly asymmetric to the mean [27]. To identify potential factors that could be predictors of anxiety and anger, stepwise linear regression analyses were performed. The results collected in both stages of the study were included in the analyses.

## 3. Results

### 3.1. Descriptive Statistics

Table 2 and Table 3 present descriptive statistics of the analyzed quantitative variables in stages I and II.

### 3.2. Level of Anxiety and Anger in Stages I and II of the Study

The level of anxiety was significantly higher in the first stage of the study compared with the next measurement after 3 months, but the effect size was small. There was no statistically significant difference in the general level of anger state. A lower level in the second stage of the study was noticed only in the anger feeling subscale, but with a very small size effect. The results are presented in Table 4.

### 3.3. Predictors of Anxiety and Anger

Due to the strong correlation between anger and anxiety (I: Pearson *r* = 0.616, *p* < 0.001, II: Pearson *r* = 0.716) models were presented in which the above-mentioned variables were not included.

The model for the subjective level of anxiety state as a dependent variable proved to be well adjusted to the data (F(14.1681) = 79.01, *p* < 0.001), explaining 39% of the dependent variable variance (adjusted *r*^2^ = 0.39). The final model with the values of standardized β coefficients is presented in Table 5.

The model for the subjective anger state level as a dependent variable proved to be well adjusted to the data (F(6.1689) = 68.04, *p* < 0.001), explaining 19% of the dependent variable variance (adjusted *r*^2^ = 0.19). The final model with the values of standardized β coefficients is presented in Table 6.

## 4. Discussion

The COVID-19 pandemic, like all epidemics, is an example of a massive situational crisis. As a result, it is a source of great stress both for individuals and social groups. The pandemic situation initiated the need to study its psychological aspects and anticipate and prevent its distant psychological consequences. Although psychological crises can manifest themselves in various ways, research indicates that anxiety and anger are the most prominent emotions during the COVID-19 pandemic, as well as other crises [28].

At the same time, however, it is known that these emotions can be used constructively and contribute to a person’s psychological development [20]. For this reason, this work aimed to show how anxiety and anger change in the initial period of the crisis. In addition, objective factors explaining these emotions’ occurrence in the medical student group were identified.

Anxiety is the primary emotion that arises in the face of danger. This is a natural reaction to an uncertain situation. At the beginning of the pandemic, at the time when the first measurement was carried out, there was a lot of misinformation and unpredictability as to the development of the situation. In our study, the participants’ average anxiety level was 50.67 points. In previous studies on cut-off points, it is assumed that the average total score above 40 on the STAI state scale corresponds to the presence of significant clinical symptoms of anxiety [29]. Medical students are generally considered to be at risk of developing anxiety disorders, at a much higher rate than the general population [30,31]. This is partly because medical education is considered to be one of the most academically and emotionally demanding training programs of any profession. This includes, for example, the difficulty of the education program and exams, contact with suffering and death, often studying outside the place of residence and the resulting cultural and language differences [32,33]. Researchers have shown that during previous epidemics, such as MERS-CoV and SARS-CoV-1, the level of anxiety among medical students was also high [34,35].

Our study showed that over the first three months of the COVID-19 pandemic crisis, the level of anxiety as a state significantly decreased in the subjects. It reached the level of 46.8 points, which, however, was still above the cut-off point for the diagnosis of the clinical level of the disorder [29]. A decrease in the level of anxiety took place although the total number of cases and deaths due to COVID-19 was steadily increasing. Similar observations were made by Daly and Robinson [36], who analyzed all sociodemographic groups, including participants with pre-existing mental health problems. They observed a sharp increase in distress in the initial phase of the COVID-19 pandemic, followed by a return to baseline within a few months. Researchers have suggested that there may be psychological resilience at the population level in response to the pandemic. Moreover, in a study by Shuster and colleagues [37], the fluctuation of depression and anxiety was explored using the COVID-19 pandemic as a model crisis. A total of 1512 adults living in the United States were surveyed and evaluated weekly for 10 weeks. It has also been shown that levels of depression and anxiety were high at the beginning but decreased over time. In a study conducted in Israel, where nursing students were examined twice, during the initial lockdown and 5 weeks later, a significant decrease in anxiety levels was similarly demonstrated [38]. It seems that the decline in the level of anxiety observed in the cited studies resulted from the gradual reduction in restrictions and misinformation over time, and the resumption of the functioning of public places, which restored the possibility of direct contact with other people. Generally, however, the mental changes that commonly occur during a crisis can be explained by the functioning of natural adaptive mechanisms that, over time, enable individuals to adapt to the situation and return to a relative emotional balance after an earlier period of mental destabilization [39].

This study showed that the strongest predictors of anxiety among the surveyed medical students were: contact with a person diagnosed or suspected of COVID-19 in the last 14 days; a high subjective assessment of the risk of the relatives becoming seriously ill in the case of a potential SARS-CoV-2 infection; and a low subjective general health assessment. All these factors are related to the direct risk of illness by the examined persons or their relatives. This is confirmed by a study conducted in April 2020 with the participation of U.S. students. It showed that the high level of anxiety of the respondents in connection with the COVID-19 pandemic was mainly due to anxiety for their health and the health of their loved ones [40]. Similarly, in studies conducted in China, it was found that during the pandemic, anxiety related to the health of oneself and family members prevailed in the general population [41].

In our study, the presence of anger was observed at the level of 27.09 points in the first measurement and 26.58 points in the second. In both cases, the results should be considered as high according to the norms [42] and in comparison with the results obtained in a similar group of students before the COVID-19 pandemic period [43]. A study conducted by Ahmed [44] and colleagues before the outbreak of the COVID-19 pandemic showed that over 90% of medical students experience anger. Stress was identified as its main predictor Taking into account the previously quoted works on the increased level of anxiety in medical students, it can be seen that both anxiety and anger are above average in this group. Studies by other authors also suggest that, especially in a crisis, anxiety and anger occur together. For example, among the residents of Salisbury, where in March 2018 there was an incident of poisoning three people with the Novichok chemical agent, any degree of anxiety, anger, and uncertainty was reported by 40.6%, 29.8%, and 30.6% of respondents, respectively [45].

The general level of the state of anger did not change among the medical students we surveyed. Only the level of feeling angry (F) decreased significantly compared with the first measurement. This could be explained by the current life circumstances of the respondents, but also from the fact of re-filling the questionnaires because the purpose and questions were already known to the students. The strongest predictor of anger turned out to be the increased need for social support in the past 14 days in the first period of the pandemic. This is an interesting result, especially in the context of the study group. It is worth mentioning here that not only was the need for support examined, but also the extent to which the support received by the respondents was sufficient. Whether or not they received adequate support did not prove to be a predictor of anger. It can therefore be assumed that the very need for support from others strengthens the feeling of anger, perhaps as a result of the accompanying feeling of helplessness and dependence on others. Observations of the authors and research by other authors show [46] that students of medical faculties can idealize their future profession in terms of desirable personal qualities, such as self-confidence, decisiveness, and independence. In this situation, even a partial loss of control over the situation and the need to ask for help can be a source of frustration and anger. Medical students may be afraid that displaying symptoms of depression or mental health problems may cause them to be perceived by others as incompetent, unprofessional, and thus unfit for their future profession [47]. Other barriers to medical students seeking help include concerns about confidentiality, time, cost, perceived stigma, potential repercussions, and fear of unwanted interventions [48,49].

Common predictors of anger and anxiety in the period of early adaptation to the COVID-19 crisis among medical students were also sought. It was shown that they have a low subjective general health assessment and a high subjective assessment of the risk that relatives will be infected by them. Similar results on anxiety have been obtained in other studies. For example, German students who were worried about being (re)infected with COVID-19 had a 1.37-fold greater risk of experiencing anxiety than those who had no such concerns. In contrast, people with pre-existing cardiovascular health conditions had an up to 3.21 times higher chance of reporting depressive symptoms [50]. Having relatives or friends infected with COVID-19 was a risk factor for increased anxiety among Chinese students [51]. There are few studies on anger during the COVID-19 pandemic. The available reviews usually focus on identifying the causes of the deterioration of general mental health and wellbeing, mentioning irrational anger or rage as one of the symptoms [52,53,54]. All the more, we believe that our study fills the gap in this area and contributes to a better understanding of the entire spectrum of emotions caused by the crisis. At the same time, it encourages the search for psychological interventions aimed at reducing the level of destructive anger, using the energy resources associated with this emotion for constructive action. Promising results in this regard come, for example, from research on mindfulness training. It has been shown that the practice of mindfulness can reduce the intensity of anger rumination, which contributes to the improvement of mental, emotional, and social wellbeing among students [55]. The positive impact of mindfulness practice on the reduction in anxiety symptoms is well documented [56,57]. A cross-sectional study of both anxiety and anger during the COVID-19 pandemic found that overall mental distress correlated positively with anxiety and anger and negatively with mindfulness. Based on the results of the multiple regression analysis, the authors of the study suggested that programs and psychological interventions promoting mental health should be designed to reduce anxiety and anger, as well as family members’ support of those infected with COVID-19 [28].

Based on the results of our study, we believe that research on anger in a crisis situation should be continued. It is an emotion less obvious in such circumstances than fear or anxiety, but it turns out that it is also present. In anger, there is a potential for energetic and motivational actions, which is why the possibility of using it to deal with a crisis seems promising. Although we do not know if there are beneficial effects of anger in different kinds of crisis situations, as well as what intensity and duration of being angry turn out to be disadvantageous and health harmful. It can be expected that in a typical adaptation to a crisis, negative emotions will drop, but it is not known what happens in situations of overlapping crises. Referring to our study and taking into account the current situation in Poland, we wonder what are the dynamics of emotional reactions and relations between them in a situation of overlapping mass situational crises such as now: the COVID-19 pandemic, the crisis with the war in Ukraine (neighboring country), and the refugee and economic crises That is why we suggest assessing such dynamics in natural conditions by taking into account changes in the scope of objective indications of the crisis and emotional responses. Monitoring the effectiveness and adequacy of social and professional support offered to young people is also important.

As academic teachers, we also believe that the results of our study should prompt consideration of updating medical curricula. In the face of massive crises, such as the COVID-19 pandemic, and individual ones, such as the illness or death of a family member, it seems reasonable for medical students to learn about the specifics of crises, their courses, and potential consequences. Moreover, they could learn how they can help people experiencing a crisis, but also how to use the potential of the crisis themselves as a factor of personal and professional development. Classes on such topics could be included in psychological modules or constitute a separate subject.

### Limitations

It was necessary to conduct this study online, which made it possible to reach numerous students in a short time and obtain relatively large research groups. However, despite efforts to eliminate selection bias, we are aware that the results obtained in two cross-sectional studies are not sufficient for unambiguous conclusions. Therefore, it would be valuable to monitor the anxiety and anger of even fewer people over a longer period and in relation to crises that are common in a given community as well as more individuals.

We did not collect enough data on psychiatric diagnoses, past psychiatric history, or psychotherapy use. The survey did not directly mention the catalog of psychiatric disorders to which the respondents could comment. However, many persons reported that they had a medical diagnosis, while not specifying it. Therefore, the number of people with mental disorders and/or a history of psychiatric treatment is difficult to determine. In the context of the purpose of the study, such data are important. They can modulate both the level of anxiety and anger in crisis situations. Therefore, in future studies, regardless of the variables related to the nature and specificity of the crisis situation that come to the fore, which may affect the mental state, it is necessary to control the variables related to the history of psychiatric treatment.

## 5. Conclusions

As a result of early adaptation to the situational crisis on the example of the COVID-19 pandemic, the general level of anxiety state and current feelings about the anger of medical students significantly decreased. The general level of anger has not changed.

The best common predictors of fear and anger during the crisis are: health self-assessment, the risk of infection in relatives, current fatigue, and websites as the main sources of knowledge about the coronavirus and the epidemic situation.

The level of anxiety is explained mostly by contact with a person infected or suspected of being infected with SARS-CoV-2 in the last 2 weeks. The level of anger is explained by the need for greater social support.

## Figures and Tables

**Table 1:** Demographic and clinical characteristics of studied groups.

Variable		Stage I n = 949		Stage II n = 748	
		N	%	N	%
Gender	Female	786	82.8	616	82.4
	Male	161	17.0	131	17.5
	No gender declaration	2	0.2	1	0.1
Partner status	In relationship	591	62.3	434	58.0
	Single	358	37.7	315	41.9
Having children	Yes	40	4.2	20	2.7
	No	909	95.8	728	97.3
Parents over 60	Yes	150	15.8	114	15.2
	No	799	84.2	634	84.8
Professional activity in the past 14 days	Yes—non-health worker	107	11.3	120	16.0
	Yes—health worker	93	0.8	107	14.3
	No	749	78.9	521	69.7
Faith	Believer	634	66.8	497	66.4
	Non-believer	315	33.2	251	33.5
Chronic diseases	Cardiological diseases	27	2.8	36	4.8
	Respiratory diseases	49	5.2	54	7.2
	Hypertension	15	1.6	12	1.6
	Diabetes	10	1.1	14	1.9
	Hypothyroidism/Hashimoto’s disease	21	2.2	14	1.9
	Allergy	6	0.6	7	0.9
	Depression	4	0.4	2	0.3
	Other (not specified)	126	13.3	105	14.0
Symptoms suggesting COVID-19 in the last 14 days	Fever > 38 °C for a min. 24 h	0	0.0	0	0.0
	Muscle aches	57	6.0	39	5.2
	Fatigue	275	29.0	246	32.9
	Sore throat	182	19.2	87	11.6
	Cough	122	12.9	53	7.1
	Dizziness	57	6	65	8.7
	Rhinitis	281	29.6	92	12.3
	Breathing problems	14	1.5	13	1.7
	Headaches	256	27.0	182	24.3
	Chills	13	1.4	13	1.7
	Lack of any symptoms	348	36.7	363	48.5

**Table 2:** Descriptive statistics of quantitative variables in stage I (n = 949).

Variable	M	Me	SD	SK	K	Min.	Max.	K-S	*P*
Anxiety state	50.67	51	11.65	−0.14	−0.53	20	79	0.06	<0.001
Anger state	27.09	24	10.37	1.05	0.68	15	60	0.12	<0.001
Feeling Angry (F)	10.74	10	3.92	0.48	−0.40	5	20	0.09	<0.001
Feeling Like Expressing Anger Verbally (V)	9.19	8	4.24	0.96	0.06	5	20	0.16	<0.001
Feeling Like Expressing Anger Physically (P)	7.16	5	3.26	1.93	4.02	5	20	0.28	<0.001
Stress self-assessment during the epidemic	3.41	4	1.05	−0.38	−0.43	1	5	0.22	<0.001
Adherence to recommendations for reducing the risk of SARS-CoV-2 infection	54.43	55	6.32	−0.97	2.28	15	65	0.08	<0.001
Number of h/24 h of staying at home due to the epidemic during the last 14 days	21.74	23	3.27	−2.82	9.84	0	24	0.28	<0.001
Correctness of predicted behavior in case of being suspected of SARS-CoV-2 infection	0.84	0.89	0.13	−1.04	2.10	0.11	1	0.21	<0.001
Subjective SARS-CoV-2 infection risk assessment	2.66	3	1.06	0.18	−0.55	1	5	0.18	<0.001
Subjective assessment of the risk of transferring infection to other people	2.87	3	1.28	0.03	−1.06	1	5	0.15	<0.001
Subjective risk of developing severe COVID-19	2.08	2	1.04	0.75	−0.13	1	5	0.22	<0.001
Subjective assessment of infection risk with SARS-CoV-2 in relatives	2.96	3	0.76	−0.05	−0.34	1	5	0.07	<0.001
Subjective assessment of the risk of relatives becoming seriously ill in case of SARS-CoV-2 infection in:									
Parents	3.46	3	0.99	−0.29	−0.23	1	5	0.20	<0.001
Grandparents	4.25	5	1.13	−1.52	1.40	1	5	0.34	<0.001
Partners	2.33	2	1.13	0.55	−0.41	1	5	0.20	<0.001
Siblings	2.32	2	1.05	0.52	−0.29	1	5	0.21	<0.001
Children	1.90	1	1.12	1.08	0.24	1	5	0.30	<0.001
Friends	2.35	2	0.93	0.29	−0.28	1	5	0.21	<0.001

M—mean; Me—median; SD—standard deviation; SK—skewness; K—kurtosis; Min. and Max.—minimum and maximum value; K–S—Kolmogorov–Smirnov test; *p*—probability.

**Table 3:** Descriptive statistics of quantitative variables in stage II (n = 748).

Variable	M	Me	SD	SK	K	Min.	Max.	K-S	*p*
Anxiety state	46.80	46	12.07	0.10	−0.62	20	77	0.07	<0.001
Anger state	26.58	24	10.51	1.02	0.56	15	60	0.14	<0.001
Feeling Angry (F)	10.26	10	3.94	0.51	−0.46	5	20	0.10	<0.001
Feeling Like Expressing Anger Verbally (V)	9.19	8	4.31	0.94	−0.06	5	20	0.17	<0.001
Feeling Like Expressing Anger Physically (P)	7.13	5	3.24	1.89	3.68	5	20	0.28	<0.001
Stress self-assessment during the epidemic	3.07	3	1.17	−0.13	−0.80	1	5	0.17	<0.001
Adherence to recommendations for reducing the risk of SARS-CoV-2 infection	51.95	53	8.37	−1	1.69	13	65	0.08	<0.001
Number of h/24 h of staying at home due to the epidemic during last 14 days	17.91	20	5.77	−1.26	1	0	24	0.22	<0.001
Correctness of predicted behavior in the case of being suspected of SARS-CoV-2 infection	0.84	0.89	0.16	−1.49	2.99	0	1	0.23	<0.001
Subjective SARS-CoV-2 infection risk assessment	2.78	3	1.13	0.07	−0.72	1	5	0.18	<0.001
Subjective assessment of the risk of transferring infection to other people	2.92	3	1.24	−0.05	−0.99	1	5	0.16	<0.001
Subjective risk of developing severe COVID-19	2.02	2	1.07	0.83	−0.15	1	5	0.24	<0.001
Subjective assessment of infection risk with SARS-CoV-2 in relatives	2.86	3	0.84	−0.11	−0.49	1	5	0.07	<0.001
Subjective assessment of the risk of relatives becoming seriously ill in case of SARS-CoV-2 infection in:									
Parents	3.34	3	1.11	−0.22	−0.58	1	5	0.18	<0.001
Grandparents	3.99	4	1.22	−1.04	0.02	1	5	0.28	<0.001
Partners	2.23	2	1.15	0.63	−0.42	1	5	0.20	<0.001
Siblings	2.32	2	1.08	0.45	−0.46	1	5	0.19	<0.001
Children	1.67	1	0.99	1.51	1.79	1	5	0.35	<0.001
Friends	2.36	2	0.98	0.30	−0.31	1	5	0.20	<0.001

M—mean; Me—median; SD—standard deviation; SK—skewness; K—kurtosis; Min. and Max.—minimum and maximum value; K-S—Kolmogorov–Smirnov test; *p*—probability.

**Table 4:** The level of anxiety and anger during stages I and II.

	Stage I n = 949		Stage II n = 748				95% CL		
	M	SD	M	SD	*t*	*p*	LL	UL	*d*
Anxiety state	50.67	11.65	46.80	12.07	6.70	0.000	2.74	5.01	0.33
Anger state	27.09	10.37	26.58	10.51	0.98	0.326	−0.50	1.50	0.05
Feeling Angry (F)	10.74	3.92	10.26	3.94	2.49	0.013	0.10	0.86	0.12
Feeling Like Expressing Anger Verbally (V)	9.19	4.24	9.19	4.31	−0.02	0.987	−0.41	0.41	0.00
Feeling Like Expressing Anger Physically (P)	7.16	3.26	7.13	3.24	0.16	0.870	−0.29	0.34	0.01

CL—confidence level; M—mean; SD—standard deviation; *t*—the value of the *t*-test statistic; *p*—probability; LL—lower limit; UL—upper limit; *d*—Cohen’s measure of sample effect size for comparing two sample means.

**Table 5:** Results of linear regression analysis for anxiety state.

Variable	B	SE	Beta	*t*	*p*
(Constant)	37.80	2.85		13.27	<0.001
Contact with a person diagnosed or suspected of COVID-19 in the last 14 days	9.35	0.51	0.37	18.51	<0.001
Subjective assessment of infection risk with SARS-CoV-2 in relatives	2.20	0.33	0.15	6.64	<0.001
Subjective general health assessment	−2.93	0.32	−0.19	−9.13	<0.001
Gender	−3.11	0.60	−0.10	−5.16	<0.001
Adherence to recommendations for reducing the risk of SARS-CoV-2 infection	0.16	0.03	0.10	4.87	<0.001
Fatigue (current symptom)	2.41	0.52	0.09	4.67	<0.001
Subjective assessment of the risk of transferring infection to other people	0.71	0.19	0.08	3.67	<0.001
Diabetes	−5.83	1.96	−0.06	−2.97	0.003
Respiratory diseases	−3.51	1.00	−0.07	−3.51	<0.001
Subjective risk of developing severe COVID-19	0.80	0.26	0.07	3.09	0.002
Parents over 60	1.85	0.64	0.06	2.91	0.004
Internet (information source about the epidemic)	1.33	0.54	0.05	2.45	0.015
Social media (information source about the epidemic)	1.07	0.46	0.04	2.33	0.020
Chills (current symptom)	−3.78	1.87	−0.04	−2.03	0.043

**Table 6:** Results of linear regression analysis for anger state.

Variable	B	SE	Beta	*t*	*p*
(Constant)	25.01	1.76		14.23	<0.001
Increased need for social support in the past 14 days	6.47	0.50	0.30	12.95	<0.001
Subjective general health assessment	−1.65	0.31	−0.13	−5.34	<0.001
Subjective assessment of infection risk with SARS-CoV-2 in relatives	1.57	0.29	0.12	5.35	<0.001
Fatigue (current symptom)	2.27	0.51	0.10	4.40	<0.001
Internet (information source about the epidemic)	1.20	0.54	0.05	2.21	0.027
Other information sources about the epidemic	1.46	0.68	0.05	2.15	0.032

## Data Availability

The data presented in this study are available on request from the corresponding author.
